# Machine Learning Methods for Prediction of CDK-Inhibitors

**DOI:** 10.1371/journal.pone.0013357

**Published:** 2010-10-13

**Authors:** Jayashree Ramana, Dinesh Gupta

**Affiliations:** Structural and Computational Biology Group, International Centre for Genetic Engineering and Biotechnology (ICGEB), New Delhi, India; Ludwig-Maximilians-Universität München, Germany

## Abstract

Progression through the cell cycle involves the coordinated activities of a suite of cyclin/cyclin-dependent kinase (CDK) complexes. The activities of the complexes are regulated by CDK inhibitors (CDKIs). Apart from its role as cell cycle regulators, CDKIs are involved in apoptosis, transcriptional regulation, cell fate determination, cell migration and cytoskeletal dynamics. As the complexes perform crucial and diverse functions, these are important drug targets for tumour and stem cell therapeutic interventions. However, CDKIs are represented by proteins with considerable sequence heterogeneity and may fail to be identified by simple similarity search methods. In this work we have evaluated and developed machine learning methods for identification of CDKIs. We used different compositional features and evolutionary information in the form of PSSMs, from CDKIs and non-CDKIs for generating SVM and ANN classifiers. In the first stage, both the ANN and SVM models were evaluated using Leave-One-Out Cross-Validation and in the second stage these were tested on independent data sets. The PSSM-based SVM model emerged as the best classifier in both the stages and is publicly available through a user-friendly web interface at http://bioinfo.icgeb.res.in/cdkipred.

## Introduction

Cyclin-dependent kinases (CDKs) are poised to play a central role in the orderly transition of the eukaryotic cells through different stages of the mitotic cell division cycle [1]. The activities of the CDKs are controlled by a tight network of regulatory mechanisms, which comprise activatory/inhibitory phosphorylation and dephosphorylation events [2], controlled degradation of the cyclin partner and association with effectors (CDK inhibitors or CDKIs) [1], [3]. Several CDKIs (such as p21, p57, p27 etc.) function as tumour supressors [4], [5], [6], [7] and loss/subversion of its activities (by mutations, elevated or decreased levels of expression etc.) results in the development of tumours, cancers and neoplasms [8], [9]. The importance of CDKIs in benign and malignant leukaemias, urological and other diseases (e.g. p57 in Beckwith-Wiedemann Syndrome) [10] is a subject of intense ongoing investigation. Though initially considered as tumour suppressors based on their ability to block cell proliferation, CDKIs play pertinent roles in the regulation of a myriad of cellular processes including transcription, apoptosis, cell migration and cytoskeletal dynamics, which may be oncogenic under certain circumstances [3], [11]. Due to the involvement of CDKs in critical cellular roles, inhibition of CDKs harbors immense relevance for anticancer therapy [11]. Inhibition of CDKs could be accomplished both by over expression of cellular CDKIs [12] as well as pharmacological inhibitors. Cellular CDKIs e.g. the tumour suppressor gene products p16^INK4^, p21^WAF1^, and p27^KIP1^, form the starting point for the design of mechanism-based CDK inhibitors [13]. Analysis of the structural aspects of cellular CDKIs leads to the identification of inhibitory lead peptides amenable to peptidomimetic development. Conversion of these peptides into pharmaceutically useful molecules provides a wealth of potential drug candidates capable of inhibiting CDKs, blocking cell-cycle progression, modulating transcription and inducing apoptosis selectively in cancer cells. Some of these, such as flavopiridol (L868275, HMR1275; Aventis), 7-hydroxystaurosporine (UCN-01, KW-2401; Kyowa Hakko Kogyo) and roscovitine (R-roscovitine, CYC202; Cyclacel), have already reached the stage of clinical evaluation [14], [15]. These pharmacological CDKIs herald the opening of new avenues of clinical therapies against such intractable pathogens like human immunodeficiency virus (HIV-1) [16] and several protozoan parasites like *Plasmodium*, *Trypanosoma* and *Leishmania* [17], [18], [19]. CDKIs also constitute potential targets for therapeutic stem cell manipulations [20].

In the light of well established significance of CDKI proteins within the cell and in the development of pharmacological CDK inhibitors, it becomes essential to have facile methods of identifying these proteins. However, these exhibit a lot of diversity in their amino acid sequences. These are represented by INK4 [21] and Cip/Kip [22] families in mammals, Sic1 protein [23] in fungi and SIAMESE (SIM) [24] family and ICK/KRP (Inhibitor of CDK/Kip-Related Protein) [25] family in plants. Owing to this enormous diversity, its identification is precluded by simple similarity-based approaches. As an alternative to similarity based methods, we applied two machine learning techniques, namely, Support Vector Machines (SVM) and Artificial Neural Networks (ANN) to address this problem. We used compositional features including amino acid composition (AAC), Split Amino Acid Composition (SAAC), Di-peptide Composition (DPC) and gapped dipeptide composition (2-gram), as well as evolutionary information from the Position-Specific Scoring Matrix (PSSM) profiles obtained from Position-Specific Iterative-Basic Local Alignment Search Tool (PSI-BLAST) for training the classifiers.

Given the immense biomedical merit and therapeutic potential of CDKIs, we anticipate that this would be a useful tool for the research community.

## Results

### Performance of alignment-based techniques

The positive training set was checked for the presence of Pfam [26] domains. The Pfam database contains three CDKI-related profiles (*CDI* (PF02234), *CDKN3* (PF05706) and *P19Arf_N* (PF07392)). It was found that only 40 out of 56 CDKI sequences showed the presence of any one of these three Pfam signatures at an E-value threshold of 1.0. Moreover, the high diversity in the sequences of CDKIs would preclude the detection of the true positives also with similarity-based searches. This was evident from our assessment of PSI-BLAST on the positive dataset in a manner similar to Leave-one-out cross-validation (LOO CV).

Three iterations of PSI-BLAST were carried out at an E-value threshold of 0.001. Each sequence was used as the query sequence once while the rest were used as the reference database and this was looped over each sequence. It was found that 10 sequences did not find any significant hit, bringing forth that general methods of similarity-based searches do not provide a reliable solution to the identification of CDKIs and a method specific to these proteins should be developed. Therefore, we set forth to explore machine-learning based methods based on various protein features for the prediction of CDKI proteins.

### Performance of alignment-free methods - SVM and ANN

#### SVM

Several SVM models were generated by varying the parameters C and γ during LOO CV, however only the best ones (as described in Methods) were selected and are depicted in Table 1. The performance measures were checked at different thresholds of SVM scores ranging from −1.0 to 1.0 and the threshold where the model yielded the best ones was used for further predictions by the model. The PSSM based model showed the best accuracy (90.44%), followed by AAC (89.88%), DPC (86.23%), SAAC (83.42%) and 2-gram composition (79.77%). The other measures (sensitivity, specificity, Matthews Correlation Coefficient (MCC), Positive predictive value (PPV) etc.) also followed the same order. Thus PSSM model emerged as the best one amongst the SVM classifiers. Moreover, the performance of the PSSM model was an improvement over the normal PSI-BLAST search (as described in the previous section), since here the sensitivity was 49/56 (87.50%) unlike PSI-BLAST (where it was 46/56, i.e. 82.14%). PSSM-based SVM classifiers are have been employed for a plethora of classification problems in biology and are well known for their remarkable performance for extremely diverse proteins like lipocalins [27], nucleic acid binding proteins [28], etc. Apart from capturing residue composition, the PSSM profiles encapsulate useful information about conservation of residues at crucial positions within the protein sequence, because in evolution the amino acid residues with similar physico-chemical properties tend to be highly conserved due to selective pressure.

**Table 1:** Performance of different SVM classifiers in LOO CV.

*Model*	*C*	*γ*	*Th*	*SN (%)*	*SP (%)*	*Accuracy (%)*	*MCC*	*PPV*
AAC	1	0.01	−0.7	87.50	90.33	89.88	0.68	0.62
SAAC	5	0.001	−0.6	83.92	83.33	83.42	0.55	0.48
DPC	0	0.01	−0.7	85.71	86.33	86.23	0.60	0.53
2-gram	0	0.01	−0.8	82.14	79.33	79.77	0.48	0.42
PSSM	5	5.00	−0.6	87.50	91.00	90.44	0.69	0.64

Th- Threshold, SN – sensitivity, SP – specificity, MCC – Matthews Correlation Coefficient, PPV- Positive predictive value.

#### Neural networks

Different ANNs were generated for the different types of features, while optimizing the learning parameters including activation function, number of hidden neurons, learning rate etc. The approach was to keep the number of hidden neurons and the number of training cycles as low as possible while simultaneously achieving good accuracies in cross-validation. Each of the neural networks had a multilayer feed forward topology. The vanilla backpropagation [29] algorithm was used to minimize the differences between the computed output and the target value. Random weights were used for initializing the net. The best ones for each feature are described below and tabulated in Table 2.

**Table 2:** Performance of best ANN classifiers in LOO CV.

*Model*	*SN (%)*	*SP(%)*	*Accuracy (%)*	*MCC*	*PPV*
AAC	67.85	88.33	85.11	0.50	0.27
SAAC	64.28	89.00	85.11	0.49	0.52
DPC	82.14	92.00	90.44	0.67	0.65
2-gram	69.64	89.66	86.51	0.54	0.27
PSSM	67.85	91.66	87.92	0.56	0.60

SN – sensitivity, SP – specificity, MCC – Matthews Correlation Coefficient, PPV- Positive predictive value.

#### AAC based ANN

This consisted of a fully-connected network, with 20 nodes in the input layer, 12 in the hidden layer and 1 in the output layer. The linear activation function was used for the input layer while the logistic function was used for the hidden and the output layer. The training was carried out for 5000 cycles and learning terminated when SSE was minimum. This yielded an accuracy of 85.11%, but sensitivity was lower (67.85%) than specificity (88.33%).

#### SAAC based ANN

This consisted of a fully-connected network, with 60 nodes in the input layer, 8 in the hidden layer and 1 in the output layer. In this case too the linear activation was used for the input layer with the logistic function for the other two layers. The training was carried out for 1000 cycles and learning terminated when SSE was minimum. This had an accuracy of 85.11%, but sensitivity (64.28%) was even lower than the AAC-based ANN, while specificity was a little higher than the latter (89%).

#### DPC based ANN

This consisted of a fully-connected network, with 400 nodes in the input layer, 2 in the hidden layer and 1 in the output layer. Here, the logistic function was used as activation function for each of the three layers. The training was carried out for 2000 cycles and learning terminated when SSE was minimum. This had an accuracy of 90.44%, and appreciably higher sensitivity (82.14%) than other ANNs with a high specificity of 92%.

#### 2-gram based ANN

This consisted of a fully-connected network, with 400 nodes in the input layer, 2 in the hidden layer and 1 in the output layer. The logistic function was used as activation function for all the three layers and the training was carried out for 1000 cycles. This ANN had a higher sensitivity (69.64%) than AAC- and SAAC- based ANNs but could not provide any improvement over the DPC-based ANN. The overall accuracy was 86.51% and specificity was 89.66%.

#### PSSM-based ANN

This consisted of a fully-connected network, with 400 nodes in the input layer, 10 in the hidden layer and 1 in the output layer. The logistic activation was used for input and output layers while linear activation function was employed for hidden layer. The training was carried out for 6500 cycles and terminated at minimal SSE. This ANN had overall accuracy of 87.92% and specificity of 91.66% with low sensitivity of 67.85% and could not provide any significant improvement over other ANNs.

Though the overall accuracy was good enough with all the ANNs, it had a higher contribution from the negative set rather than the positive set, so the DPC-based ANN emerged as the most efficient classifier which had good sensitivity as well as specificity.

### Performance on benchmark datasets

We tested the performance of the best classifiers only on the benchmark datasets (i.e. Datasets S3 and S4). Table 3 depicts the sensitivity, specificity and PPV of these classifiers on these two datasets. Herein, we observed better prediction efficiency for the DPC-based SVM model over the AAC-based SVM model, unlike that in cross-validation. It is plausible that some over-fitting of data could have occurred in cross-validation with AAC. The DPC-based ANN also performed almost at par with the SVM models. As expected, the PSSM-based SVM model yielded the best performance amongst all the tested classifiers on all the test sets.

**Table 3:** Performance on benchmark datasets.

*Model*	*Positive set (48)*	*Negative set (308)*	*PPV*
SVM-AAC	38 (79.16)	274 (88.96)	0.52
SVM-DPC	45 (93.75)	285 (92.53)	0.66
SVM-PSSM	47 (97.91)	294 (95.45)	0.77
ANN-DPC	42 (87.50)	277 (89.93)	0.57

PPV- Positive Predictive Value. The numbers show the correctly predicted sequences out of the total shown in the first row, 48 for the positive set and 308 for the negative set. The sensitivity and specificity percentages are reported within the brackets in the second and third columns respectively.

Based on the performance in LOO CV and on benchmark datasets, the PSSM-based SVM model was selected as the best model. Its performance was further tested on randomly picked up phosphatases and kinases (Datasets S5 and S6). A number of phosphatases and kinases bind to CDKs as do the CDKIs. Moreover a CDK inhibitor i.e. Cyclin-dependent kinase inhibitor 3 (CDKN3) or Kinase-associated phosphatase (KAP), is a dual-specificity phosphatase which contains the HCXX-XXGR motif characteristic of protein tyrosine phosphatases [30]. This protein binds to CDK2 and dephosphorylates Thr160 when the associated cyclin subunit is degraded or dissociates, thereby rendering CDK2 inactive [31], [32]. Hence phosphatases and kinases are potent candidates for false positive predictions.

Amongst the 65 kinases, two were wrongly predicted as CDKIs while amongst the 65 phosphatases, four were wrongly predicted as CDKIs by the PSSM-based SVM model. Thus the false positive prediction rate of the model is low enough to justify its utility for practical applications.

### Receiver Operating Characteristic (ROC) plot

**Figure 1:**
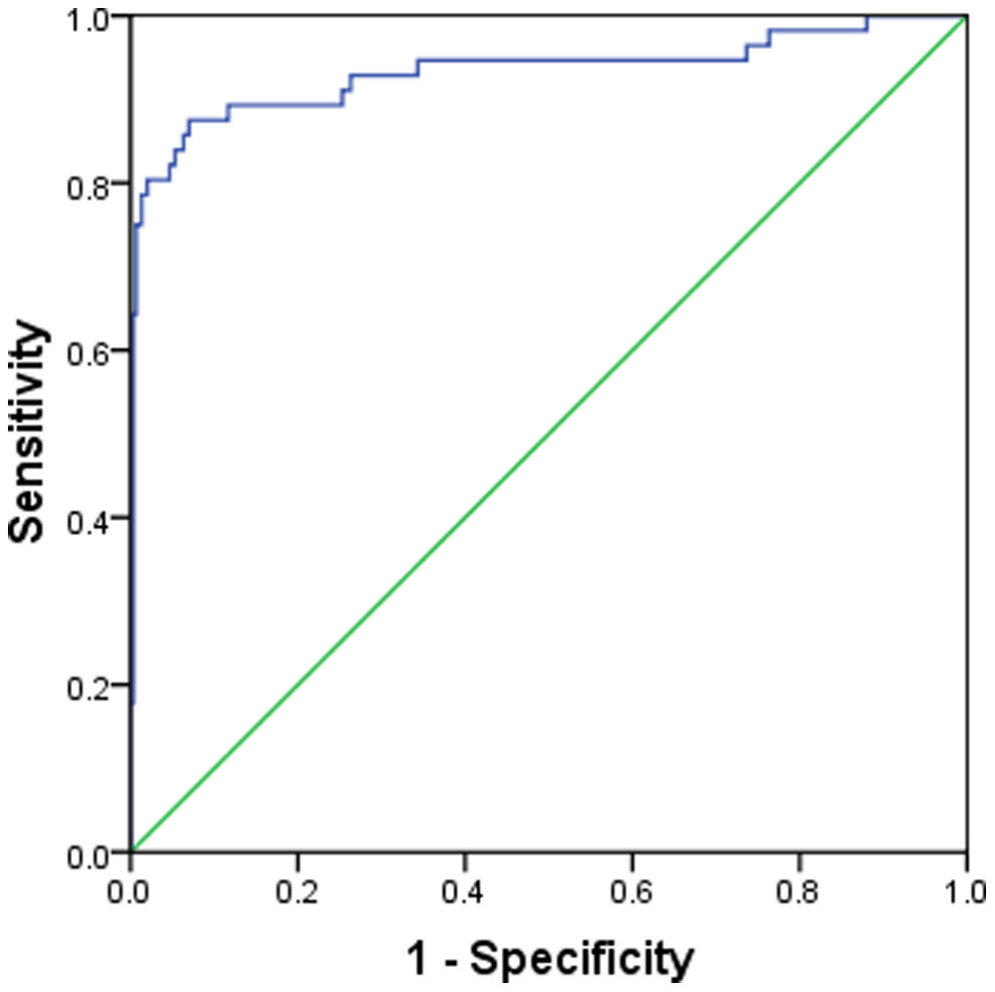
ROC plot of PSSM-based SVM model. The ROC curve depicts relative trade-offs between true positive and false positives. The green line is the reference line while the blue curve represents the ROC curve for the PSSM-based SVM model with an Area Under Curve (AUC) of 0.933.

ROC curves show the trade-off between true positive rate (sensitivity) and false positive rate (1- specificity) over their entire range of possible values. It is considered as the most robust approach for classifier evaluation [33]. The Area Under Curve (AUC) is used as a reliable index of classifier performance. This validates the threshold-independent performance of the classifiers. We plotted the ROC curve for the best classifier, PSSM-based SVM model (Figure 1). The curve had an AUC of 0.933 which further reinforced the discriminative efficiency of the model.

### Web implementation

**Figure 2:**
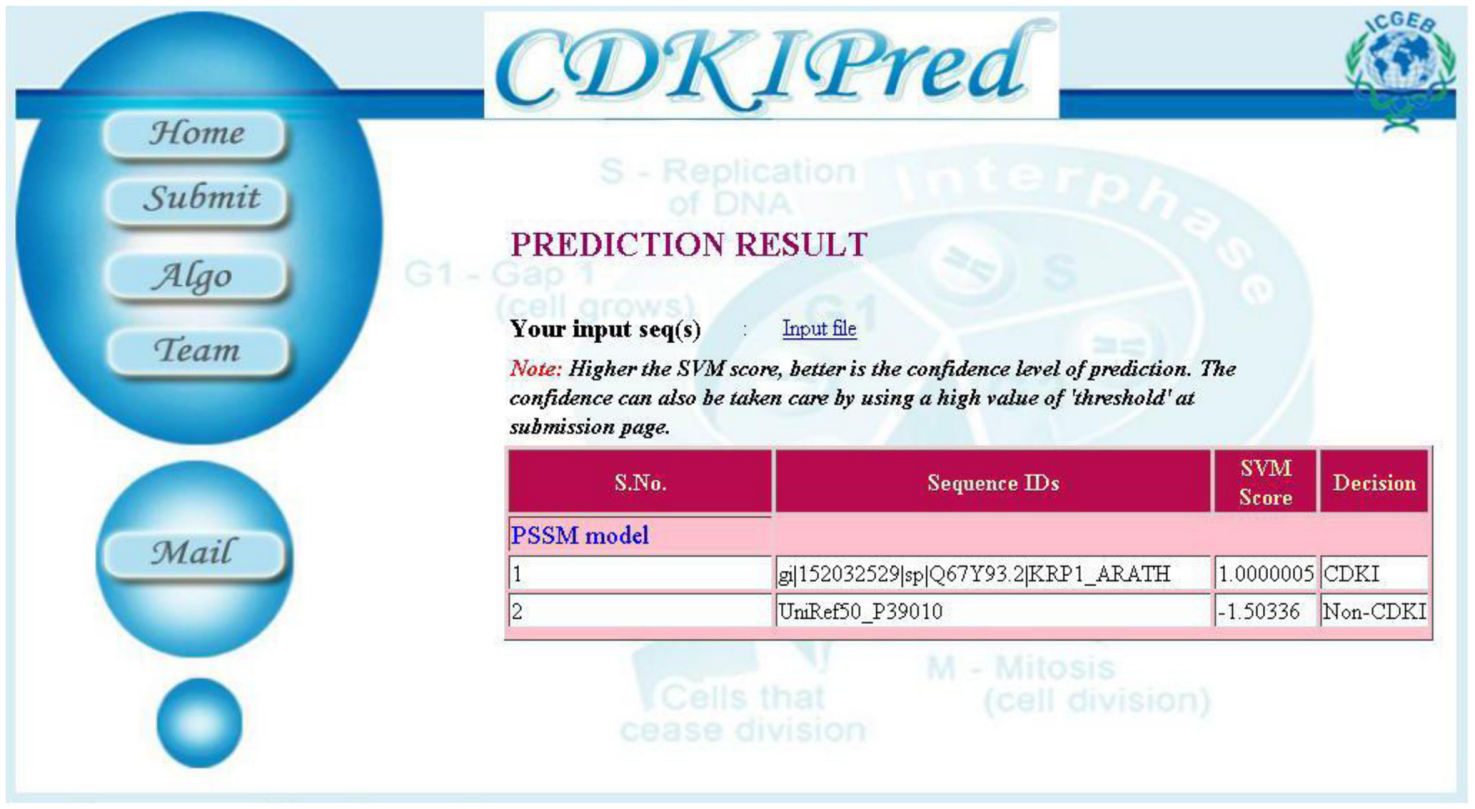
Snapshot of web server sample output. The web server predicts CDKIs based on the best classifier, i.e. PSSM-based SVM model. The server accepts FASTA formatted sequences and allows user defined thresholds of prediction, ranging from −1.0 to 1.0.

The Web server was developed using Apache (version 2.0). Server side scripting was done in PHP (version 5.0). The prediction algorithm presented in this study is implemented as a freely accessible web server at http://bioinfo.icgeb.res.in/cdkipred (Figure 2). The program predicts CDKI sequences using the SVM model based on PSSM profile. The input sequences are provided in the FASTA format. The program allows the users to perform prediction at thresholds ranging from −1.0 to 1.0 for SVM score. The program output returns the sequence ID, the SVM score and the decision of the classifier regarding the sequence based on the threshold chosen.

## Discussion

This is the first report of a machine-learning-based method for identification of CDKI protein sequences. Previously, such approaches have been applied to the computational identification of other components of the cell cycle including cyclins [34] and CDK phosphorylation substrates [35].

Our tool simply represents a complementary tool to allow the detection of CDK inhibitors, since we prove that PFAM signatures miss a significant number of the already known CDK inhibitors from the non-redundant set. However such machine learning based methods do come at the cost of some false positive predictions, which should be as minimal as possible. We tested this on an independent dataset comprising of randomly picked up non-CDKIs as well as kinases and phosphatases which are the most likely candidates for false positive predictions and indeed obtained a low false positive prediction rate.

In this study, we observed that SVM based methods are more efficient than ANN in discrimination of CDKI and non-CDKI sequences, despite the imbalance in the size of the positive and the negative training datasets (56 and 300 respectively). This was observed with all the types of features (Table 1 and Table 2). For an experimenter, a judicious approach would be minimizing the number of CDKIs to be characterized by increasing the threshold to higher SVM score, in order to get only the topmost candidates for further work. Supplementing these with other complementary evidence like domain knowledge and sub-cellular localization may provide inroads to the discovery of novel CDKIs and further our understanding of cell cycle regulation and other cellular phenomena. In future, the availability of more sequences and inclusion of more features may further enhance the prediction accuracy.

## Methods

### Generation of datasets for SVM and ANN training

Two sets containing CDKI and non-CDKI sequences were compiled from different databases- including Genbank, SwissProt and RefSeq. The positive set was prepared through keyword search using keywords like ‘CDK inhibitor’ while the negative set was assembled by randomly picking the non-CDKIs. Both the sets were manually inspected to prevent the mislabeling of the data. The redundancy in both the sets was removed at a similarity threshold of 40% using the CD-HIT program [36]. This yielded a positive set of 56 well-annotated and mostly experimentally verified CDKIs (Dataset S1) and a negative set of 300 non-CDKIs (Dataset S2).

### Benchmark dataset for testing

In order to gauge the utility of our best SVM models and ANNs for unseen sequences, we tested their performance on independent datasets not used in training or testing cycles. While one test dataset consisted of 48 CDKIs (Dataset S3), the other had 308 non-CDKIs (Dataset S4). Moreover, we prepared two datasets for testing the performance of the best model in the study. These consisted of randomly picked up sequences of 65 phosphatases (Dataset S5) and 65 kinases (Dataset S6) from SwissProt.

### Training features used as input for SVMs and NNs

Different compositional features, i.e. AAC, SAAC, DPC and 2-gram composition were extracted from the positive and negative dataset sequences. Evolutionary information in the form of PSSM profiles for both the sets was also used to train SVM models and ANNs.

AAC is the fraction of each of the 20 amino acids present in a protein sequence. This generates an input vector of 20 dimensions. For calculating SAAC, the protein was split into three parts of equal length and the fraction of each amino acid calculated separately for the three. This generates an input vector of 60 dimensions. DPC is the fraction of a dipeptide divided by the total number of possible dipeptides in the given protein sequence. This yields a training vector of 400 dimensions. 2-gram composition is the fraction of dipeptides composed by two amino acids with one separating residue in between. This yields a training vector of 400 dimensions. The PSSM was obtained by performing PSI-BLAST against SwissProt database (release 57.13) at the E-value threshold of 0.001 with three iterations. The matrix contains 20×N elements, N being the length of the query sequence, and each element represents the frequency of a particular residue substitution at a specific position in the alignment. To generate input vectors of fixed length for SVM training, the PSSM matrix was normalized between 0 and 1 using the following logistic function:
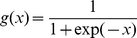
Where x is the raw value in PSSM profile and g(x) is the normalized value of x. Following this, the normalized matrix is organized into a composition matrix of fixed length pattern of 400 (20×20, for each amino acid, there are 20 substitution scores from normalized matrix).

### SVMs and SVM^light^

SVM is a supervised machine learning method extensively used in classification and regression problems based on Structural Risk Minimization (SRM) principle from statistical learning theory [37]. SVM is used in conjunction with kernel functions which implicitly map input data to a higher dimensional non-linear feature space. SVM builds a model (classifier) by constructing an optimal hyperplane that divides the positively and negatively labeled samples with the maximum margin of separation. To construct an optimal hyperplane, SVMs employ an iterative training algorithm, which is used to minimize an error function. Hyperplanes are searched in the space of possible inputs; subsequently these hyperplanes are used to separate positive and negative patterns. The selected data points supporting the hyperplane are called support vectors.

We implemented SVM using the software SVM^light^ written and distributed by Joachims [38]. This package enables users to select from a choice of inbuilt kernel functions and to define a number of parameters for each kernel function. For a given kernel function, a large number of models (classifiers) can be built by varying the input values for its parameters and evaluated. We used Radial Basis Function (RBF) kernel to train and test our training datasets. The values of γ and regularization parameter C were optimized on the training datasets by cross-validation. The approach was aimed at choosing the parameters so as to maximize accuracy along with nearly equal sensitivity and specificity, wherever possible.

### ANN and SNNS

The Artificial Neural Network (ANN) consists of nodes or neurons that receive signals through interconnecting arcs [39]. Signals are passed between neurons (organized in input, hidden and output layers) through connection links which carry an associated weight. Each neuron applies a non-linear transformation called an activation function to its net input to determine it output signal.

We used the freely available package Stuttgart Neural Network Simulator (http://www.ra.cs.uni-tuebingen.de/SNNS/), SNNS version 4.2 to implement ANN. One advantage that this package offers is that it allows the incorporation of the trained networks in ANSI C functions for use in stand-alone code. The feed-forward back propagation type of neural networks was trained on different protein features. The number of hidden nodes and other learning parameters were optimized for each network. The output unit consisted of target value 1 or 0, referring to positives and negatives respectively. The Sum of Squared Error function (SSE) on training was monitored after every training cycle. The final number of cycles was determined where the SSE was the least. The value of the learning rate was set to 0.1.

### Leave-One-Out Cross -Validation (LOO CV)

This is considered as the most objective and rigorous mode of evaluation wherein one dataset sequence is kept for testing, while the rest are used to train the model/NN. This is repeated till each sequence becomes the testing data exactly once. This is a stringent case of n-fold cross-validation where n equals the total number of sequences. The various performance measures (explained below) are taken for n folds and then averaged to get overall assessment of the classifier.

### Classifier performance metrics

To evaluate the accuracy of SVM classifiers and ANNs developed in cross-validation cycles; we used four measures. *Sensitivity* is defined as the percentage of CDKI protein sequences that are correctly predicted as CDKIs. *Specificity* is the percentage of non-CDKI protein sequences that are correctly predicted as non-CDKIs. *Accuracy* is the percentage of correct predictions out of total number of predictions. *Matthews correlation coefficient (MCC)* is a measure of both sensitivity and specificity, MCC = 0 indicates completely random prediction, while MCC = 1 indicates perfect prediction. Positive predictive value (PPV) is the likelihood that a sequence reported by the classifier as CDKI is really CDKI.
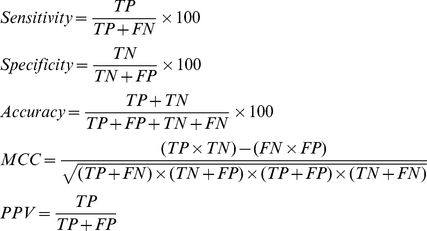



### Receiver Operating Characteristic (ROC) plot

Statistical Package for Social Sciences (SPSS v16.0) for Windows was used to obtain the ROC plot [40] for the best classifier obtained in the study.

## Supporting Information

Dataset S1 Set of 56 annotated and mostly experimentally verified CDKIs.
(0.02 MB TXT)

Dataset S2 Set of 300 non-CDKI sequences.
(0.20 MB TXT)

Dataset S3 Sequences of 48 CDKI sequences not used in the training/testing.
(0.01 MB TXT)

Dataset S4 Sequences of 308 non CDKI sequences not used in the training/testing.
(0.14 MB TXT)

Dataset S5 Phosphatase sequences used for evaluation of false positives.
(0.03 MB TXT)

Dataset S6 Kinase sequences used for evaluation of false positives.
(0.04 MB TXT)
